# Supplementary figures and images for: Prevalence and incidence of diabetic peripheral neuropathy in Latin America and the Caribbean: A systematic review and meta-analysis
Source: PLoS One. 2021 May 13;16(5):e0251642. doi: 10.1371/journal.pone.0251642 (PMC8118539; doi:10.1371/journal.pone.0251642)

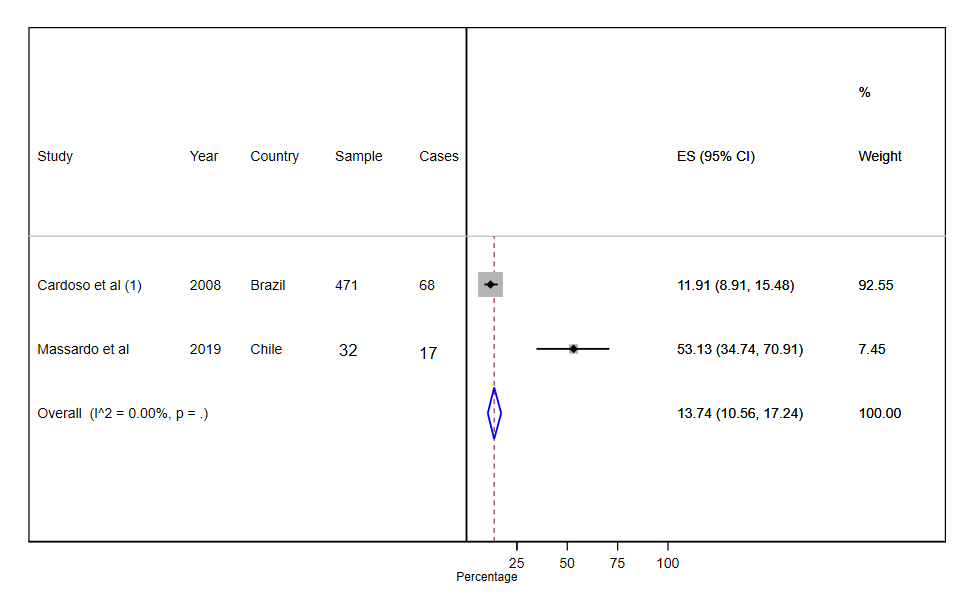

Supplement: S1 Fig — (TIF) [file pone.0251642.s002.tif]

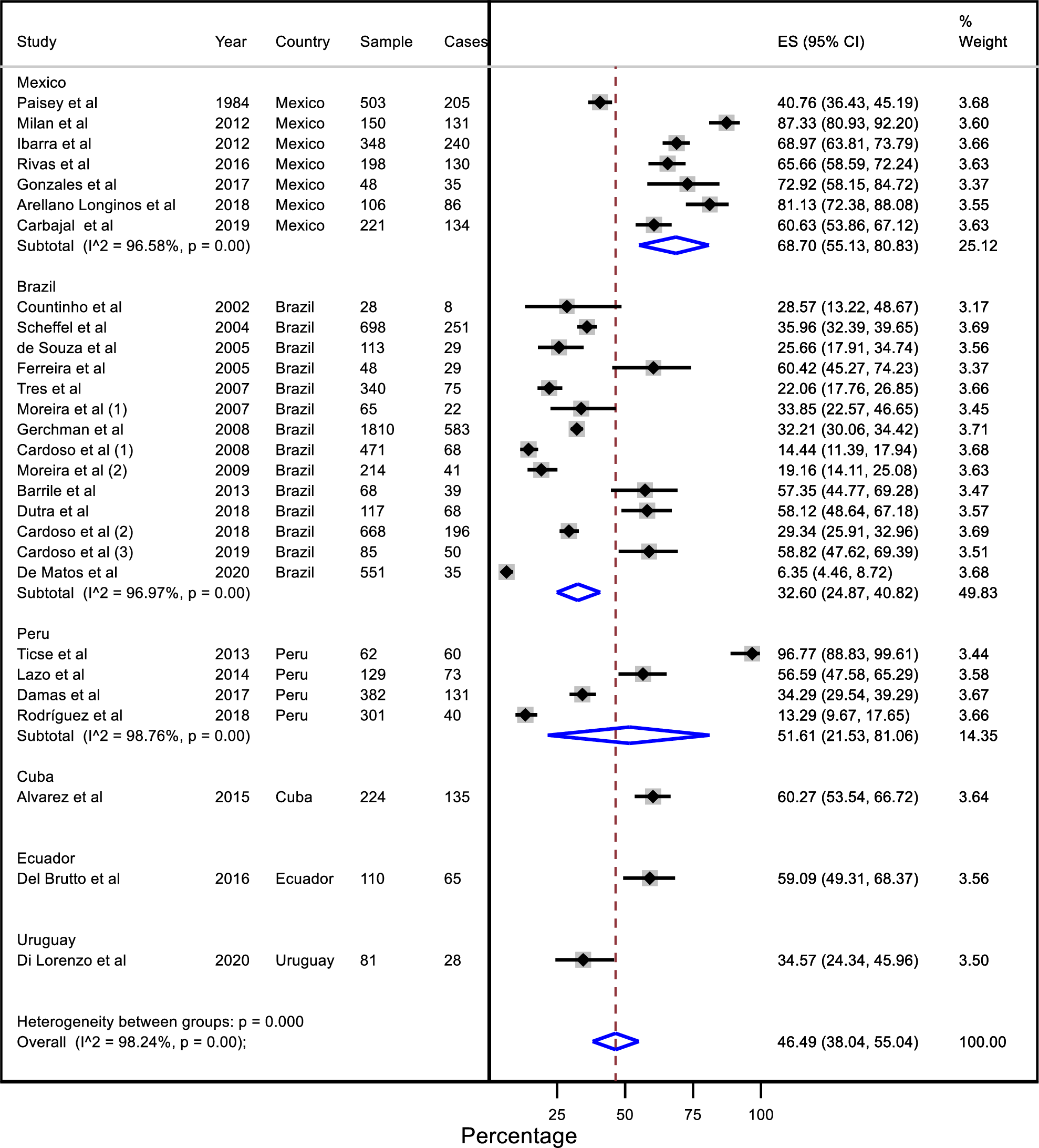

Supplement: S2 Fig — (TIF) [file pone.0251642.s003.tif]

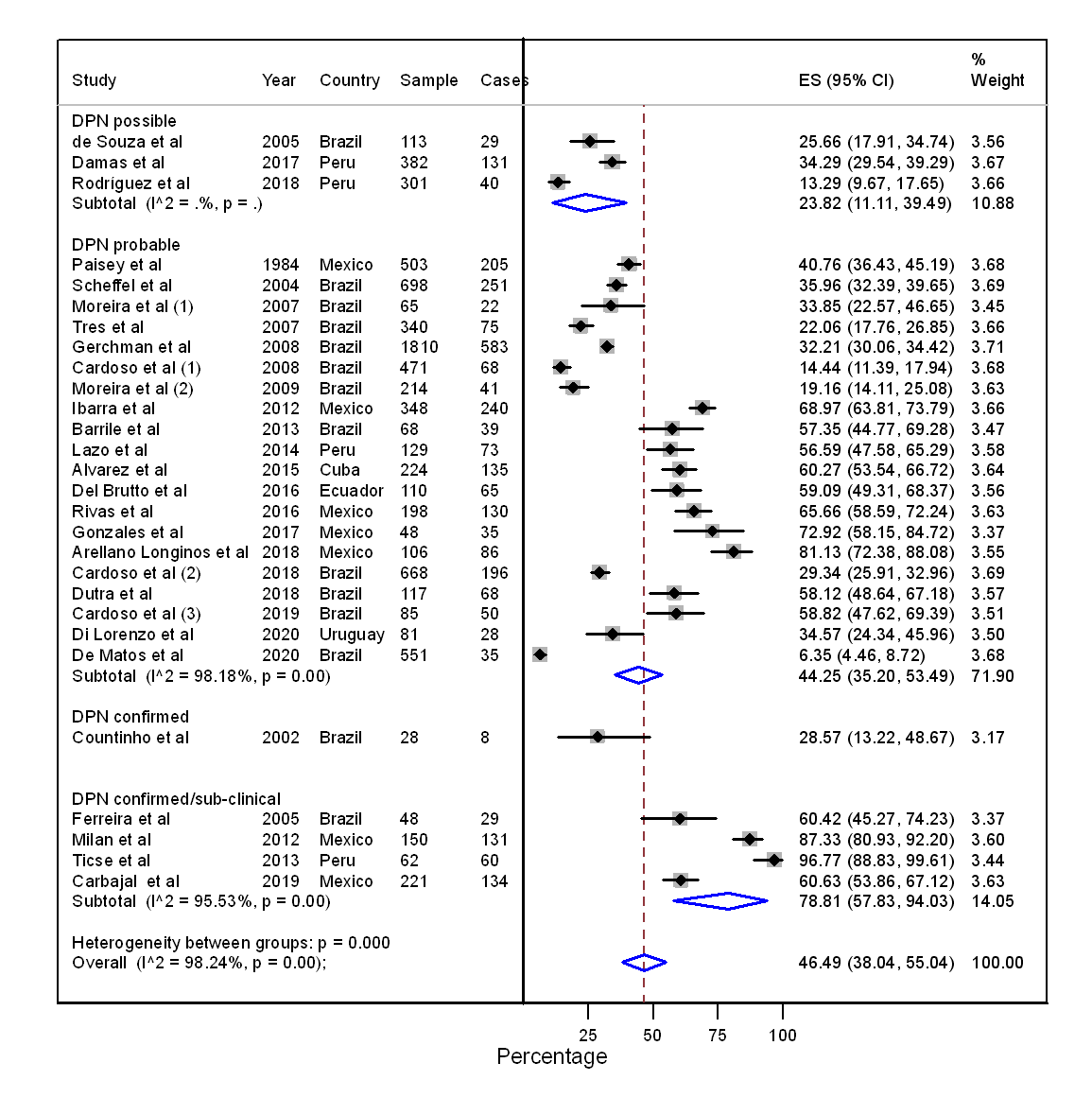

Supplement: S3 Fig — (TIF) [file pone.0251642.s004.tif]
