# Supplementary material for: Prevalence and incidence of diabetic peripheral neuropathy in Latin America and the Caribbean: A systematic review and meta-analysis
Source: PLoS One. 2021 May 13;16(5):e0251642. doi: 10.1371/journal.pone.0251642 (PMC8118539; doi:10.1371/journal.pone.0251642)
Supplement: S1 Table — (DOCX) [file pone.0251642.s005.docx]

**S1 Table. Search strategy.**

|  |  | Date | Results | Date | Results |
| --- | --- | --- | --- | --- | --- |
| Pubmed | #1 "Risk Factors"[Mesh] or “risk factor”[tiab] OR “risk factors”[tiab] OR “risks factor”[tiab] OR “risk factors”[tiab] OR “population at risk”[tiab] OR “population at risks”[tiab] OR “populations at risk”[tiab] OR “populations at risks”[tiab]  #2 prevalence[mesh] OR incidence[mesh] OR Cross-Sectional Studies[mesh] OR Epidemiology[mesh] OR Cohort Studies[mesh] OR Case-Control Studies[mesh] OR morbidity[mesh] OR Epidemiologic Studies[mesh] OR Longitudinal Studies[mesh] OR "Observational Study"[PT] OR prevalenc*[tiab] OR incidenc*[tiab] or prevalenc*[tiab] OR incidenc*[tiab] OR “cross-sectional”[tiab] OR epidemi*[tiab] OR cohort[tiab] OR “case-control”[tiab] OR “case-controls”[tiab] OR morbidit*[tiab] OR longitudinal*[tiab] OR frequenc*[tiab] OR surveillance*[tiab] OR occurrence*[tiab] OR outbreak*[tiab] OR endemic*[tiab] OR transvers*[tiab] OR prospective*[tiab] OR retrospective*[tiab] OR observation*[tiab] OR analys*[tiab] OR “follow-up”[tiab] OR “follow-ups”[tiab] or "Observational Study"[tiab] OR "Observational Studies”[tiab]  #2 Diabetic neuropathies[Mesh] or Diabetic Foot[mh] OR (Diabetic*[tiab] and (Neuropath*[tiab] or neuralgia*[tiab] or Mononeuropath*[tiab] or Polyneuropath*[tiab] or asymmetric[tiab] or symmetric*[tiab] or simpl*[tiab] or amyotroph*[tiab] or Foot[tiab]))  #4 Latin America[Mh] OR "Latin America"[tiab] OR Caribbean Region[Mh] OR "Caribbean Region"[tiab] OR South America [Mh] OR "South America”[tiab] OR Indians, South American [Mh] OR Hispanoamerica*[tiab] OR Iberoamerica*[tiab] OR Panamerican*[tiab] or Argentina[Mh] OR Argentina[tiab] OR Argentin*[ad] OR Bolivia[Mh] OR Bolivia[tiab] OR Bolivia[ad] OR Brazil[Mh] OR Brazil[tiab] Brazil*[ad] OR Brasil*[ad] OR Chile[Mh] OR Chile[tiab] OR Colombia[Mh] OR Colombia[tiab] OR Colombia[ad] OR Costa rica[Mh] OR Costa rica[tiab] or Costa Ric*[ad] OR Cuba[Mh] Or Cuba[tiab] OR Ecuador[Mh] OR Ecuador[tiab] OR Ecuador*[ad] OR El salvador[Mh] OR "El salvador"[tiab] or "El salvador"[ad] OR Guatemala[Mh] OR Guatemala[tiab] or Guatemala[ad] OR Haiti[Mh] OR Haiti[tiab] OR Honduras[Mh] OR Honduras[tiab] OR Mexico[Mh] OR Mexico[tiab] or Mexico[ad] or Mejico[ad] OR Nicaragua[Mh] OR Nicaragua[tiab] OR Panama[Mh] OR Panama[tiab] OR Paraguay[Mh] OR Paraguay[tiab] OR Paraguay[ad] OR Peru[Mh] OR Peru[tiab] OR Peru*[ad] OR Puerto Rico[Mh] OR "Puerto Rico"[tiab] OR "Puerto Rico"[ad] OR Dominican Republic[Mh] OR "Dominican Republic"[tiab] or "Dominican Republic"[ad] OR Uruguay[Mh] OR Uruguay[tiab] OR Uruguay[ad] OR Venezuela [Mh] OR Venezuela [tiab] or Venezuela [ad] OR Suriname[Mh] or Suriname[tiab] or Surinam*[ad] or Guiana*[tiab] OR Guiana*[ad] OR Guyan*[tiab] OR Guyan*[ad]  (((#1 OR #2) AND #3) AND #4) | Sep 2019 | 360 | Dec 2020 | 21 |
| Scopus | #1 ( TITLE-ABS-KEY ( prevalence OR incidence OR epidemiology OR cohort OR morbidity OR ocurrence* OR outbreak* OR retrospective* OR prospective* OR transvers* OR endemic* OR surveillance* ) ) OR ( TITLE-ABS-KEY ( studie* ) AND TITLE-ABS-KEY ( cohort OR "case control" OR observational OR epidemiolog* OR longitudinal OR "cross sectional" ) )  #2 TITLE-ABS-KEY (risk* w/1 factor*) or TITLE-ABS-KEY(population* w/1 risk*)  #3 ( TITLE-ABS-KEY ( diabetic ) AND TITLE-ABS-KEY ( neuropath* OR neuralgia* OR mononeuropath* OR polyneuropath* OR asymmetric OR symmetric* OR simpl* OR amyotroph* OR foot ) ) OR ( ( TITLE-ABS-KEY ( diabetic* AND neuropath* ) AND TITLE-ABS-KEY ( pain OR autonomic OR peripheral ) ) )  #4 ( TITLE-ABS-KEY ( argentina OR bolivia OR brazil OR chile OR colombia OR "Costa Rica" OR cuba OR ecuador OR "El Salvador" OR guatemala OR haiti OR honduras OR mexico OR nicaragua OR panama OR paraguay OR peru OR "Puerto Rico" OR "Dominican Republic" OR uruguay OR venezuela OR "Latin America" OR "South America" OR iberoamerica OR caribbean OR suriname ) OR AFFILCOUNTRY ( argentina OR bolivia OR brazil OR chile OR colombia OR "Costa Rica" OR cuba OR ecuador OR "El Salvador" OR guatemala OR haiti OR honduras OR mexico OR nicaragua OR panama OR paraguay OR peru OR "Puerto Rico" OR "Dominican Republic" OR uruguay OR venezuela OR suriname ) )  (((#1 OR #2) AND #3) AND #4) | Sep 2019 | 753 | Dec 2020 | 85 |
| WOS | #1 (TI=(DIABETIC) AND TI=(neuropath* or neuralgia* or mononeuropath* or polyneuropath* or asymmetric or symmetric* or simp* or amyotroph* or foot)) OR (TI=(DIABETIC and neuropath*) AND TI=(pain OR autonomic OR peripheral ))  #2 (AK=(DIABETIC) AND AK=(neuropath* or neuralgia* or mononeuropath* or polyneuropath* or asymmetric or symmetric* or simp* or amyotroph* or foot)) OR (AK=(DIABETIC and neuropath*) AND TI=(pain OR autonomic OR peripheral ))  #3 (KP=(DIABETIC) AND KP=(neuropath* or neuralgia* or mononeuropath* or polyneuropath* or asymmetric or symmetric* or simp* or amyotroph* or foot)) OR (AK=(DIABETIC and neuropath*) AND AK=(pain OR autonomic OR peripheral ))  #4 (TS=(DIABETIC FOOT) AND TS=(Prevalence or incidence or “risk factors”)) OR (TS=(DIABETIC AND NEUROPATH*) AND TS=(prevalence OR incidence OR “risk factors”))  #5 TS=(“risk factors” OR “population at risk”) or TS=(“risk factors” OR “population at risk”) or TS=(“risk factors” OR “population at risk”)  #6 TI=(argentina OR bolivia OR brazil OR chile OR colombia OR "Costa Rica" OR cuba OR ecuador OR "El Salvador" OR guatemala OR haiti OR honduras OR mexico OR nicaragua OR panama OR paraguay OR peru OR "Puerto Rico" OR "Dominican Republic" OR uruguay OR venezuela OR "Latin America" OR "South America" OR iberoamerica OR caribbean OR suriname) OR AK=(argentina OR bolivia OR brazil OR chile OR colombia OR "Costa Rica" OR cuba OR ecuador OR "El Salvador" OR guatemala OR haiti OR honduras OR mexico OR nicaragua OR panama OR paraguay OR peru OR "Puerto Rico" OR "Dominican Republic" OR uruguay OR venezuela OR "Latin America" OR "South America" OR iberoamerica OR caribbean OR suriname) OR KP=(argentina OR bolivia OR brazil OR chile OR colombia OR "Costa Rica" OR cuba OR ecuador OR "El Salvador" OR guatemala OR haiti OR honduras OR mexico OR nicaragua OR panama OR paraguay OR peru OR "Puerto Rico" OR "Dominican Republic" OR uruguay OR venezuela OR "Latin America" OR "South America" OR iberoamerica OR caribbean OR suriname) OR CU=(argentina OR bolivia OR brazil OR chile OR colombia OR "Costa Rica" OR cuba OR ecuador OR "El Salvador" OR guatemala OR haiti OR honduras OR mexico OR nicaragua OR panama OR paraguay OR peru OR "Puerto Rico" OR "Dominican Republic" OR uruguay OR venezuela OR "Latin America" OR "South America" OR iberoamerica OR caribbean OR suriname)  (((#1 OR #2 OR #3 OR #4) AND #5) AND #6)) | Sep 2019 | 214 | Dec 2020 | 14 |
| Scielo | The same of WOS | Sep 2019 | 99 | Dec 2020 | 5 |
| Embase | #1 DIABETIC.ab,kw,sh,ti.  #2 (neuropath* or neuralgia* or mononeuropath* or polyneuropath* or asymmetric or symmetric* or simp* or amyotroph* or foot).ab,kw,sh,ti.  #3 #1 AND #2  #4 (prevalence or incidence or epidemiology or cohort or morbidity or ocurrence* or outbreak* or retrospective* or prospective* or transvers* or endemic* or surveillance* or "risk factor*").ab,kw,sh,ti.  #5 #3 AND #4  #6 (argentina or bolivia or brazil or chile or colombia or "Costa Rica" or cuba or ecuador or "El Salvador" or guatemala or haiti or honduras or mexico or nicaragua or panama or paraguay or peru or "Puerto Rico" or "Dominican Republic" or uruguay or venezuela or "Latin America" or "South America" or iberoamerica or caribbean or suriname).ab,cp,kw,sh,ti.  #7 #5 AND #6 | Sep 2019 | 476 | Dec 2020 | 21 |
