# Supplementary material for: Prevalence and incidence of diabetic peripheral neuropathy in Latin America and the Caribbean: A systematic review and meta-analysis
Source: PLoS One. 2021 May 13;16(5):e0251642. doi: 10.1371/journal.pone.0251642 (PMC8118539; doi:10.1371/journal.pone.0251642)
Supplement: S2 Table — (DOCX) [file pone.0251642.s006.docx]

**S2 Table. Studies that were evaluated in full-text and were excluded.**

|  | Author | Year | Title | Reason for exclusion |
| --- | --- | --- | --- | --- |
| 1 | Alayón | 2009 | Chronic complications, hypertension and obesity in diabetic patients living in Cartagena, Colombia | Unclear diagnostic criteria. Data obtained from medical records. |
| 2 | Alonso | 2017 | Electrophisiological Assessment of Diabetic Patients | Different population: all patients have diabetic neuropathy |
| 3 | Alvarez | 2019 | Vitamin B12 deficiency and diabetic neuropathy in patients taking metformin: a cross-sectional study | Different population: prediabetes patients |
| 4 | Bañuelo | 2013 | Risk factors of foot ulceration in patients with Diabetes Mellitus type 2 | Insufficient diagnosis criteria. Only includes evaluation with monofilament. |
| 5 | Bezerra | 2020 | Clinical and epidemiological differences in diabetes: A cross-sectional study of the Brazilian population compared with the French GERODIAB cohor | Unclear diagnostic criteria |
| 6 | Bonilla | 2019 | Demographic and clinical characteristics of dominican adults admitted to a diabetic foot clinic in the Dominican Republic, 2015 | Unclear diagnostic criteria |
| 7 | Brinati | 2017 | Prevalence and factors associated with peripheral neuropathy in individuals with diabetes mellitus | Insufficient diagnosis criteria. Only includes evaluation with monofilament |
| 8 | Calsolari | 2002 | Retrospective Analysis of Diabetic Feet from Patients Followed in The Diabetes Clinic at Santa Casa of Belo Horizonte, MG. | Insufficient diagnosis criteria. Only includes evaluation with monofilament. |
| 9 | Cardoso | 2015 | Increased aortic stiffness predicts future development and progression of peripheral neuropathy in patients with type 2 diabetes: The Rio De Janeiro type 2 diabetes cohort study. | It´s a salami study by an author already included |
| 10 | Cardoso | 2018 | Long-term visit-to-visit glycemic variability as predictor of micro- and macrovascular complications in patients with type 2 diabetes: The Rio de Janeiro Type 2 Diabetes Cohort Study. | It´s a salami study by an author already included |
| 11 | Carrasco | 2020 | Clinical-biochemical characteristics of type 2 diabetic patients from the Ecuadorian social security Institute of Pastaza, Ecuador | Unclear diagnostic criteria |
| 12 | Carvalho | 2019 | Comparison of neurosensory devices in detecting cutaneous thresholds related to protective sensibility: A cross-sectional study in São Paulo, Brazil. | Not outcome of interest. Only shows prevalence on new method |
| 13 | Chevtchouk | 2017 | Ankle-brachial index and diabetic neuropathy: study of 225 patients. | Insufficient diagnosis criteria. Only includes pain assessment |
| 14 | Cortez | 2014 | Prevalence of neuropathic pain and associated factors in diabetes mellitus type 2 patients seen in outpatient setting | Insufficient diagnosis criteria. Only includes pain assessment |
| 15 | Elgart | 2014 | Association between socioeconomic status, type 2 diabetes and its chronic complications in Argentina. | No outcome of interest: only grouped microcomplications |
| 16 | Escobar | 2005 | Utilidad de la electroneuromiografía para evaluar neuropatía en pacientes diabéticos | Different population: Diabetes referred for electroneuromyography. |
| 17 | Espin | 2010 | Factores de riesgo asociados a neuropatía diabética dolorosa | Different population: all patients have diabetic neuropathy |
| 18 | Ferreira | 2020 | Competitive neural layer-based method to identify people with high risk for diabetic foot | Unclear diagnostic criteria |
| 19 | Ferreira | 2019 | Foot function and strength of patients with diabetes grouped by ulcer risk classification (IWGDF) | No outcome of interest: foot funcion |
| 20 | Gagliardino | 2001 | Evaluation of the quality of care for diabetic patients in Latin America | Unclear diagnostic criteria: diagnosis by self-report or by MNSI |
| 21 | Gomez Viera | 2001 | Factores de riesgo de la neuropatía diabética simétrica distal | Unclear diagnostic criteria: Doesn't define physical examination performed and type of EMG |
| 22 | Kosiborod | 2018 | Vascular complications in patients with type 2 diabetes: Prevalence and associated factors in 38 countries (the DISCOVER study program). | Different population: Countris different of LAC |
| 23 | Mejías | 2018 | Prevalence of peripheral arterial disease among diabetic patients in Santo Domingo, Dominican Republic and associated risk factors | Insufficient diagnosis criteria. Only includes evaluation with monofilament |
| 24 | Milan Guerrero | 2011 | Asociación entre la presencia de enfermedad vascular periférica y neuropatía en pacientes con diabetes mellitus tipo 2 | It´s a salami study by an author already included |
| 25 | Molina | 2020 | Peripheral neuropathy of the lower limbs in patients with type 2 diabetes mellitus | Insufficient diagnosis criteria. Only includes pain assessment |
| 26 | Muniz | 2003 | Neuropathic and Ischemic Changes of the Foot in Brazilian Patients with Diabetes | Insufficient diagnosis criteria. |
| 27 | Osuna | 2014 | Characterization of type 2 diabetes mellitus and metabolic control in the hospitalized patient | Unclear diagnostic criteria: self-report diagnosis in old patients and monofilament in new patients |
| 28 | Ovalle-Luna | 2019 | Ovalle-Luna, O. D., et al. (2019). "Prevalence of complications of diabetes and associated comorbidities in family medicine of the Mexican Institute of Social Security. | No outcome of interest: only grouped microcomplications |
| 29 | Pop-Busui | 2009 | Pop-Busui, R., et al. (2009). "Prevalence of diabetic peripheral neuropathy and relation to glycemic control therapies at baseline in the BARI 2D cohort. | Different population: Countris different of LAC |
| 30 | Ramos | 2014 | Results of the epidemiological surveillance of diabetes mellitus in hospitals in Peru, 2012 | Unclear diagnostic criteria: Data obtained from medical records. |
| 31 | Reis | 2020 | Demographic and socioeconomic profiles of patients admitted with diabetic foot complications in a tertiary hospital in Belem – Para | Not outcome of interest. Diabetic foot |
| 32 | Rolim | 2008 | Heterogeneidade clínica e coexistência das neuropatias diabéticas: diferenças e semelhanças entre diabetes melito tipos 1 e 2 | Different population: all patients have diabetic neuropathy |
| 33 | Saban-Ruiz | 2006 | Complicaciones crónicas en la diabetes mellitus. Prevalencia en una unidad de medicina familiar | Unclear diagnostic criteria: Data obtained from medical records. |
| 34 | Salama | 2001 | Factores de riesgo y complicaciones crónicas en el diagnóstico reciente de la diabetes tipo 2 | Unclear diagnostic criteria |
| 35 | Santos | 2014 | Microvascular complications in type 2 diabetes and associated factors: a telephone survey of self-reported morbidity | Unclear diagnostic criteria: diagnosis by telephone self-report |
| 36 | Scain | 2018 | Effects of nursing care on patients in an educational program for prevention of diabetic foot | Insufficient diagnosis criteria. Only includes evaluation with monofilament. |
| 37 | Toloza | 2020 | COP Analysis in Type 2 Diabetics with Peripheral Diabetic Neuropathy: (Open Data May Contribute with Prognosis and Intervention in Early Stages to Reduce a Risk of Falling | Case control study |
| 38 | Udall | 2019 | Epidemiology of physician-diagnosed neuropathic pain in Brazil. | Insufficient diagnosis criteria. Only includes pain assessment |
| 39 | Untiveros | 2012 | Late Complications in type 2 Diabetes Mellitus at the Hospital II Essalud – Cañete | Unclear diagnostic criteria |
| 40 | Villegas | 2004 | Control y complicaciones crónicas de la diabetes mellitus en el Centro de Atención Ambulatorio central, Instituto de Seguro Social 1998-2001 | Unclear diagnostic criteria: Data obtained from medical records. |
| 41 | Zerquera | 2016 | Caracterización de los pacientes diabéticos tipo 2 ingresados en el Centro de Atención al Diabético de Cienfuegos | Unclear diagnostic criteria: The Dick criteria used are not the actual Dick criteria.. |

MNSI: Michigan Neuropathy Screening Instrument; EMG: Electromyography
