# Supplementary material for: Prevalence and incidence of diabetic peripheral neuropathy in Latin America and the Caribbean: A systematic review and meta-analysis
Source: PLoS One. 2021 May 13;16(5):e0251642. doi: 10.1371/journal.pone.0251642 (PMC8118539; doi:10.1371/journal.pone.0251642)
Supplement: S3 Table — (DOCX) [file pone.0251642.s007.docx]

**S3 Table. Quality assessment of prevalence studies according Loney Scale for cross-sectional studies.**

| Study (year) | Q1: Is the design and sampling method appropriate for the question? | Q2: Is the sampling frame appropriate? | Q3: Adequate sample size (> 323 subjects)? | Q4: Was the diagnositc criteria validated? | Q5: Was the result measured in an impartial way (without bias)? | Q6: Do the interviewees match the target population (Response> 70%.) | Q7: Were estimates given with confidence intervals or a subgroup used? | Q8: Is the target population described? | Total |
| --- | --- | --- | --- | --- | --- | --- | --- | --- | --- |
| Alvarez et al 2015 | YES | YES | NO | YES | NO | NO | NO | YES | 4 |
| Arellano Longinos et al 2018 | YES | YES | NO | YES | NO | YES | NO | YES | 5 |
| Barrile et al 2013 | NO | NO | NO | YES | NO | NO | NO | YES | 2 |
| Carbajal et al 2019 | NO | NO | NO | YES | NO | NO | YES | YES | 3 |
| Cardoso et al (2) 2018 | NO | NO | YES | YES | YES | NO | YES | YES | 5 |
| Cardoso et al (1) 2008 | NO | NO | YES | YES | YES | NO | YES | YES | 5 |
| Cardoso et al (3) 2020 | NO | NO | NO | YES | NO | YES | YES | YES | 5 |
| Countinho et al 2002 | NO | NO | NO | YES | YES | NO | YES | YES | 4 |
| Damas et al 2017 | YES | YES | YES | YES | NO | YES | YES | YES | 7 |
| Di Lorenzo et al 2020 | NO | NO | NO | YES | NO | YES | YES | YES | 5 |
| de Souza et al 2005 | YES | YES | NO | YES | YES | YES | YES | YES | 7 |
| De Matos 2020 | NO | NO | YES | YES | NO | YES | YES | YES | 5 |
| Del Brutto et al 2016 | YES | YES | YES | YES | YES | YES | YES | YES | 8 |
| Dutra et al 2018 | NO | NO | NO | YES | YES | NO | YES | YES | 4 |
| Ferreira et al 2005 | NO | NO | NO | YES | YES | NO | YES | YES | 4 |
| Gerchman et al 2008 | YES | YES | YES | YES | NO | YES | YES | YES | 7 |
| Gonzales et al 2017 | NO | NO | NO | YES | YES | NO | YES | YES | 4 |
| Ibarra et al 2012 | YES | YES | YES | YES | YES | YES | YES | YES | 8 |
| Lazo et al 2014 | NO | NO | NO | YES | YES | NO | YES | YES | 4 |
| Milan et al 2012 | NO | NO | NO | YES | YES | NO | YES | YES | 4 |
| Moreira et al (2) 2009 | NO | NO | NO | YES | YES | NO | YES | YES | 4 |
| Moreira et al (1) 2007 | NO | NO | NO | YES | YES | NO | YES | YES | 4 |
| Paisey et al 1984 | YES | YES | YES | YES | YES | YES | YES | YES | 8 |
| Rivas et al 2016 | NO | NO | NO | YES | NO | NO | YES | YES | 3 |
| Rodríguez et al 2018 | NO | NO | NO | YES | NO | NO | YES | YES | 3 |
| Scheffel et al 2004 | YES | YES | YES | YES | NO | YES | YES | YES | 7 |
| Ticse et al 2013 | YES | YES | NO | YES | YES | NO | YES | YES | 6 |
| Tres et al 2007 | NO | NO | YES | YES | YES | NO | YES | YES | 5 |
