# Supplementary material for: Prevalence and incidence of diabetic peripheral neuropathy in Latin America and the Caribbean: A systematic review and meta-analysis
Source: PLoS One. 2021 May 13;16(5):e0251642. doi: 10.1371/journal.pone.0251642 (PMC8118539; doi:10.1371/journal.pone.0251642)
Supplement: S4 Table — (DOCX) [file pone.0251642.s008.docx]

**S4 Table. Quality assessment of incidence study according to New Castle Ottawa scale for cohort studies.**

| Study (year) | Representativeness of the exposed cohort | Selection of the non exposed cohort | Ascertainment of exposure | Demonstration that outcome of interest was not present at start of study | Comparability of Cohorts on the Basis of the Design or Analysis | Assessmnt of outcome | Was follow-up long enough for outcomes to occur | Adequacy of follow up of cohorts | Total |
| --- | --- | --- | --- | --- | --- | --- | --- | --- | --- |
| 1. Cardoso et al (2008) | YES | NO | YES | YES | NO | YES | YES | YES | 6 |
| 2. Massardo et al (2019) | YES | NO | YES | YES | NO | YES | YES | YES | 6 |

reported; DM: Diabetes Mellitus; DM2: type 1 Diabetes Mellitus; TCNS: Toronto Clinical Neuropathy Score; NO : New Castle Ottawa
